# Lymphoepithelial Carcinoma of the Breast

**DOI:** 10.7759/cureus.42597

**Published:** 2023-07-28

**Authors:** Mamta Arora, Balaji Balasubramanian, Naemieh Mohammad Kamel Aljasem, Simran Arora, Ravi Arora

**Affiliations:** 1 Department of Surgery, NMC Specialty Hospital, Abu Dhabi, Abu Dhabi, ARE; 2 Department of Surgery and Oncology, NMC Specialty Hospital, Abu Dhabi, Abu Dhabi, ARE; 3 Department of Pathology, NMC Royal Hospital, Abu Dhabi, ARE; 4 Department of Surgery, University of Debrecen, Debrecen, HUN; 5 Department of Internal Medicine and Endocrinology, NMC Specialty Hospital, Abu Dhabi, Abu Dhabi, ARE

**Keywords:** frozen section, simple mastectomy, immunohistochemistry staining, pathological diagnosis, primary breast malignancy, lymphoepithelial carcinoma

## Abstract

Lymphoepithelial malignancy is an extremely rare carcinoma of the breast characterized by a confusing histopathological picture resembling medullary carcinomas, lymphoma, etc. It has also been reported in other regions of the body like salivary glands, nasopharyngeal area and sometimes the lung. Due to its rare presence and difficult diagnosis, the treatment is often prolonged and delayed.

Here we present a case report of a 56-year-old lady who was eventually diagnosed as lymphoepithelial carcinoma of the breast. Her journey of evaluation and treatment was fraught with pathological nuances and an elimination drill of multiple differentials before concluding this rare diagnosis. Although lymphoepithelial-like carcinoma is a rare entity, multiple cases have been reported in the literature and their review is mandated to further our clinical knowledge about the oncological treatment and expected prognosis of such cases in the future. Our patient underwent a simple mastectomy, followed by chemotherapy, radiotherapy, and is completely asymptomatic now. She has been cancer-free for the last seven years so far.

## Introduction

Lymphoepithelial carcinoma is a rare malignancy reported in the nasopharynx. A similar lymphoepithelial-like carcinoma (LELC) has been reported in the breast, and there are a countable number of cases reported thus far [1]. Histopathologically, epithelial/undifferentiated cells are seen in single or multiple sheets along with numerous lymphoid infiltrates, much like the picture of nasopharyngeal carcinoma. It has been confused with lymphoma, inflammatory breast disease and sarcoidosis in the past due to its atypical picture [2].

In the lung and nasopharynx, it has been associated with Epstein-Barr virus, and human papillomavirus leading to squamous cell carcinomas with lymphoid elements [3].

## Case presentation

We present the case of a 50-year-old postmenopausal Asian lady with complaints of a left breast lump noted for three months, that had recently increased in size. The patient reported no family history of breast cancer or related cancers, was a lifelong non-smoker and teetotaler, and had no other comorbid illnesses. There was no recent history of viral infections or chest or throat symptoms. 

Clinical examination revealed a tense, firm and non-tender lump in the left breast. The lump measured about 5 x 4 cm in size was fixed and immobile in relation to surrounding breast structures, but with no visible skin involvement. Axillary examination picked a 2 x 2 cm ipsilateral axillary lymph node. The other breast and axilla were normal.

**Figure 1:**
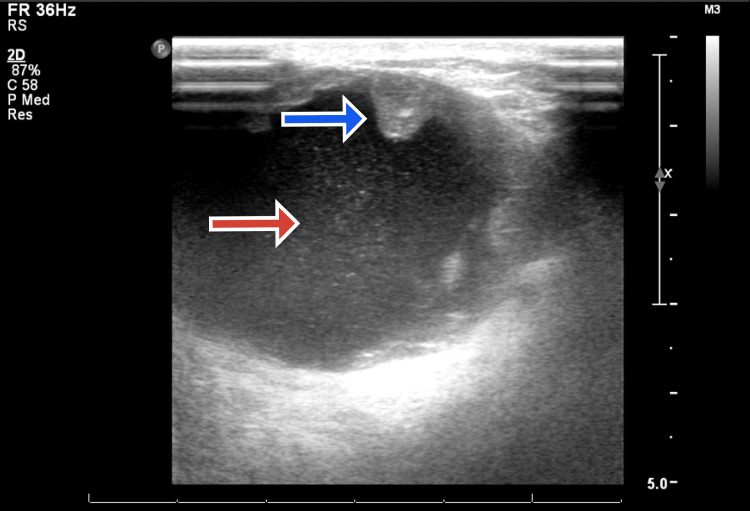
Ultrasound showed a hypoechoic area with discrete areas of calcifications with a rim of peripheral enhancement. Blue arrow shows the solid component in a non-dependent position suspended in the cyst shown by the red arrow.

Standard patient evaluation was undertaken. Ultrasound showed a large complex cystic lesion with multiple intra-cystic solid echogenic nodular components which were quite vascular in the left breast (reported BIRADS 4) (Figure 1).

**Figure 2:**
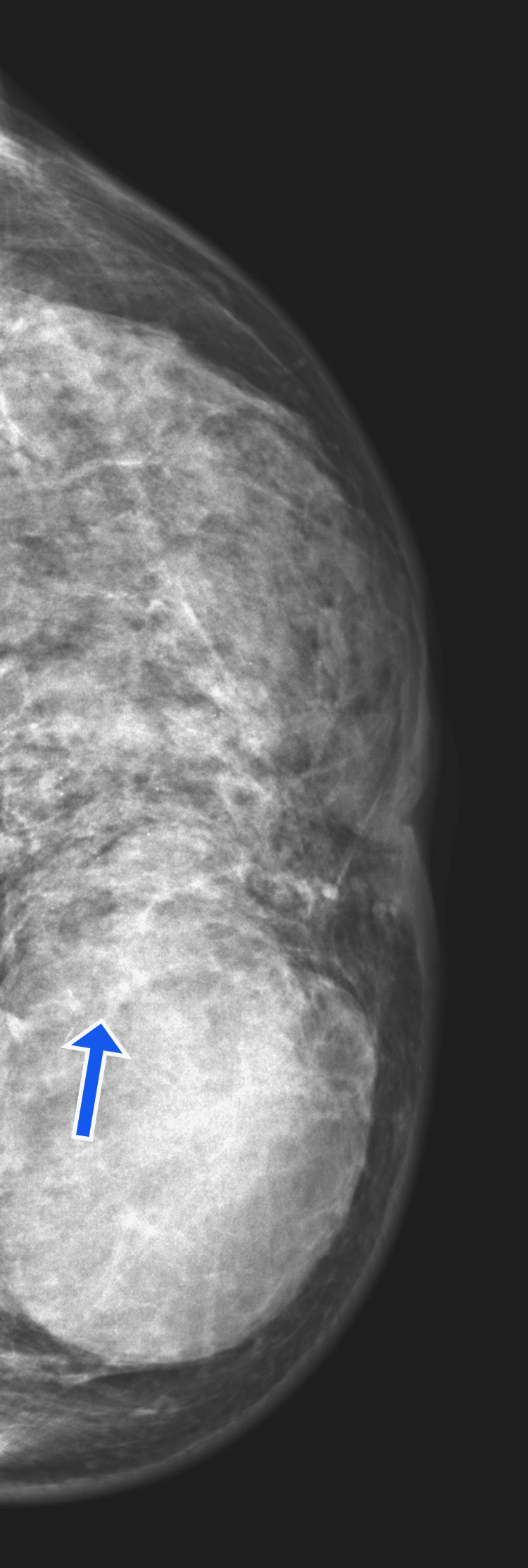
The mammogram showed a large opaque shadow in the lower inner quadrant of the breast with the blue arrow showing discrete calcifications.

The mammogram showed a large lobulated radiopaque lesion measuring 4.4 x 3 cm in the left breast's lower inner quadrant. Multiple scattered specks of calcification were noted in the left breast (BIRADS 4) (Figure 2).

The patient underwent scan-guided fine needle aspiration cytology (FNAC) from the solid portions of the complex cyst and the axillary node. Core needle biopsy was not performed owing to the largely cystic nature of the swelling. A lump was reported as suspicious for malignancy, node being negative.

**Figure 3:**
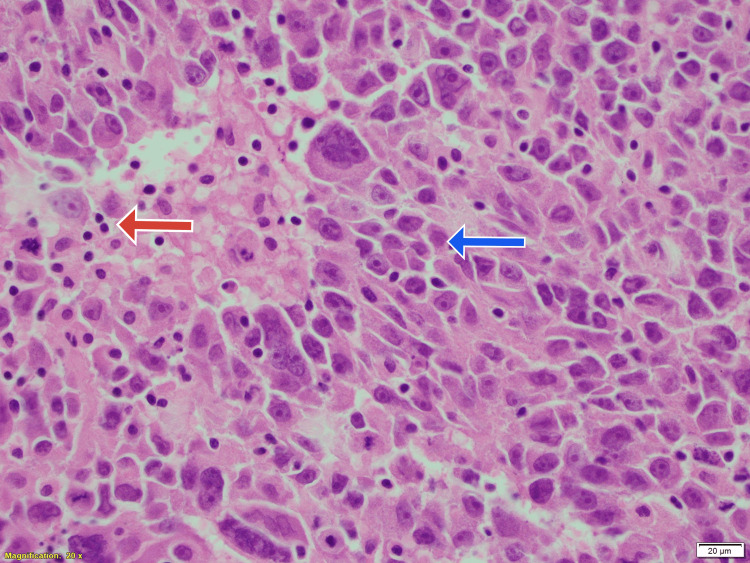
Lumpectomy specimen shows rounded cells (blue arrow) with lymphocytic predominance (red arrow). Hematoxylin and eosin stain, magnification X400.

**Figure 4:**
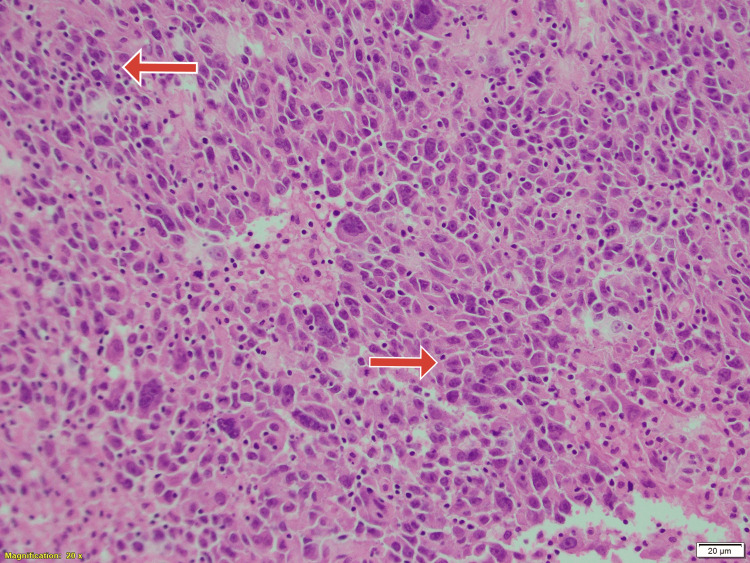
Histopathological picture of the lumpectomy specimen showing lymphocytic predominance with rounded cells (red arrows), hematoxylin and eosin stain, magnification X200.

**Figure 5:**
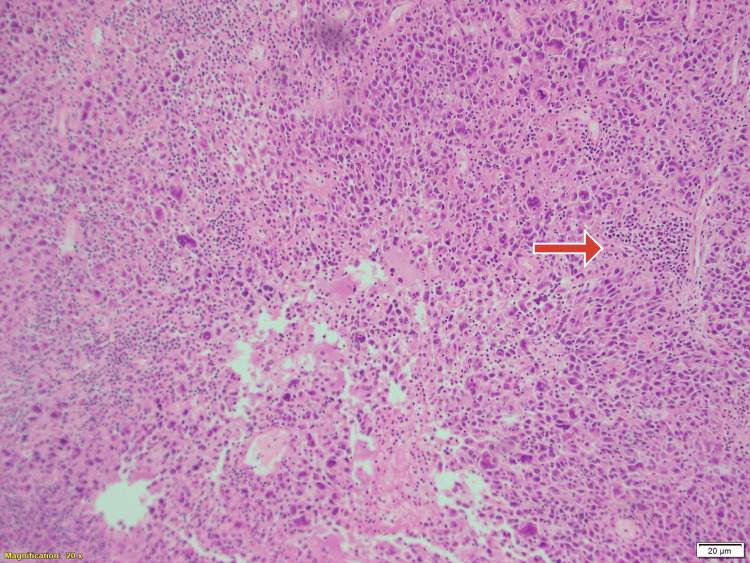
There was a dense inflammatory infiltrate of mature round lymphocytes with occasional plasma cells, histiocytes and eosinophils. Hematoxylin and eosin stain, magnification X100. Features were suggestive of lymphoepithelial-like tumour of the breast.

The rest of the investigations were normal. The patient was subsequently taken up for lumpectomy with sentinel node biopsy. A frozen section was done intraoperatively with a plan to proceed with mastectomy if malignancy was confirmed. The frozen section gave a confounding picture, looking suspicious for a lymphoma due to the lymphoid domination of the tissue, hence only lumpectomy with sentinel node biopsy was done. Histopathology of the specimen however showed sheets of large cells with abundant pale cytoplasm, large vesicular nuclei, prominent nucleolus and a pushing pattern at the margins of the tumour. There was a dense inflammatory infiltrate of mature round lymphocytes and lymphoid follicles with occasional plasma cells, histiocytes and eosinophils at the periphery. Margins seemed to be involved with the same changes (Figures 3-5).

All these findings were suggestive of a lymphoepithelial-like tumour of the breast. The lymph node was negative for malignancy.

**Figure 6:**
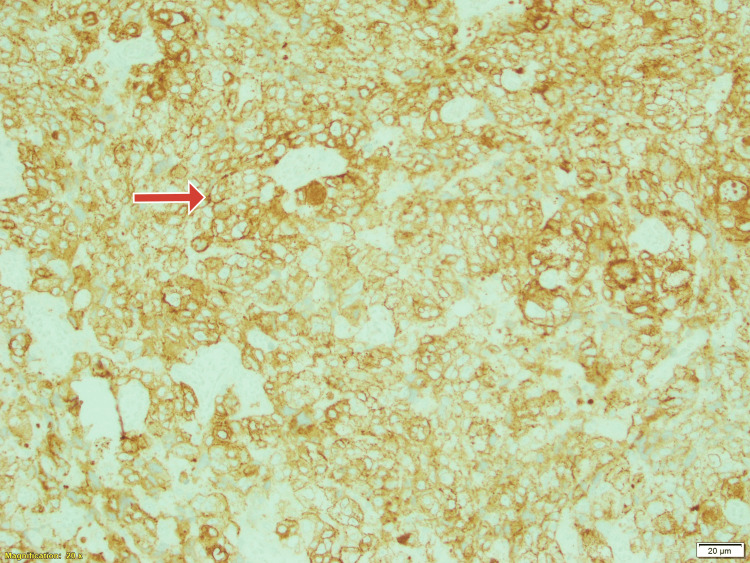
Immunohistochemistry clinched the diagnosis. Tumour was Cytokeratin positive as shown by the brown staining (red arrow). Magnification X200.

Close differentials were lymphoma and medullary carcinoma. Immunohistochemistry was done to clinch the diagnosis of lymphoepithelioma of the breast. PanCk was positive and CD-45 was positive in the lymphoid admixture. Synaptophysin showed faint scattered positivity (Figure 6).

The patient underwent a simple mastectomy at a later sitting. The removed specimen showed a residual tumour, but the margins were free. The tumour was noted to be triple negative for ER/PR/HER2. Ki67 was 90 to 95%.

The surgery was followed by chemotherapy Adriamycin (dose of 60 mg per meter square), cyclophosphamide (600 mg per meter square) four cycles followed by Taxol (weekly 80 mg per meter square for 12 weeks) between May 2016 and November 2016. The patient was given external beam radiation therapy in January 2017. She also received a zoledronic acid infusion of 4 mg once in six months for osteoporosis.

The patient has since been under our regular follow-up from 2017 till to date. No augmentation procedures were done for the breast, as per the patient's wish. She later underwent thyroidectomy for a large multinodular goitre in April 2020 but has been breast cancer-free ever since.

## Discussion

Lymphoepithelial tumour is a rare masquerading tumour, not more than 50 cases have been reported in the literature so far (Table 1).

**Table 1:** Table summarizing main clinicopathological parameters of lymphoepithelioma-like cancer of the breast cases reported so far in world literature. EBV: Epstein-Barr virus, Her2: human epidermal growth factor receptor 2

Number	Authors and reference	Year	Patient age (years)	Tumour size (cm)	Lymph node	ER (%)	PR (%)	Her2	CK AE1/AE3	EBV
1	Kumar and Kumar [1]	1994	65	2.0	0	+	+	−	+	−
2	Cristina et al. [4]	2000	54	1.5	0/19	+ (42)	− (<10)	−	NA	−
3	Dadmanesh et al. [5]	2001	43	1.9	1/1	−	−	−	NA	−
4			53	2.0		−	−	−	NA	−
5			49	1.0	0/19	−	−	−	NA	−
6			52	2.7	0/20	+	−	−	NA	−
7			64	2.0	0/29	−	−	−	NA	−
8			69	2.3	0/19	−	−	−	NA	−
9	Naidoo and Chetty [6]	2001	50	2.5	2/24	NA	NA	NA	−	−
10	Pestereli et al. [7]	2002	56	2.0	2/27	+	+	−	+	−
11	Sanati et al. [8]	2004	62	3.0	NA	+ (10)	−	−	+	−
12	Ilvan et al. [9]	2004	59	3.5	0/20	+	+	−	+	−
13			67	1.1	0/16	−	−	−	+	−
14	Kurose et al. [10]	2005	47	2.8	0/33	−	−	+	+	−
15	Saleh et al. [11]	2005	51	1.3	1/8	−	−	NA	+	−
16	Kulka et al. [12]	2008	42	2.5	0/10	+	−	−	+	−
17	O’Sullivan-Mejia et al. [13]	2009	55	3.1	0/2	−	−	+	+	−
18	Jeong et al. [14]	2010	37	3.0	0/13	−	−	+	+	−
19	Dinniwell et al. [15]	2012	55	4.0	0/2	−	−	−	NA	−
20	Nio et al. [2]	2012	45	3.0	0/5	−	−	−	NA	NA
21	Suzuki et al. [16]	2012	64	2.1	3/23	−	−	+	NA	NA
22	Trihia et al. [17]	2012	53	1.5	2/30	−	−	+	+	NA
23	Abdou and Asaad [18]	2014	45	2.0	0/24	−	−	−	NA	−
24	Top et al. [19]	2014	59	3.0	0/23	−	−	−	NA	−
25	Nankin et al. [3]	2015	39	2.7	0/5	+ (40)	−	−	NA	NA
26	Sato et al. [20]	2016	50	1.2	1/23	−	−	−	+	−
27	Herrera-Goepfert et al. [21]	2016	57	4.0	0	+	+	−	+	−
28	Shet et al. [22]	2016	56	3.0	1/17	−	−	−	NA	−
29			39	2.0	0/18	−	−	−	NA	−
30			40	2.5	NA	−	−	−	NA	−
31			40	3.5	NA	−	−	−	NA	−
32			51	3.0	NA	−	−	−	NA	−
33	Tarek et al. [23]	2017	62	3.5	0/11	−	−	−	+	NA

Lymphoepithelial carcinomas are rarely seen in the breast. It presents usually as a cystic or solid tumour. A mammogram may show a high-density irregular or poorly defined mass with or without calcifications. In sonograms, solid or cystic hypoechoic masses with micro-lobulated margins or subtle abnormal parenchyma with discrete calcifications may be seen. However, unlike classical ductal or lobular breast carcinoma, histopathology depicts sheets of epithelial cells with a prominent lymphoid infiltrate (Table 1) [23]. In nasopharyngeal and salivary glands, it has been reported as undifferentiated cells with lymphoid infiltrates. Also reported variously in the lung [24], kidney, and large bowel [25].

LELC of the breast needs to be distinguished from breast neoplasms with prominent lymphoid infiltration, such as medullary carcinoma [8], lymphoma or lymphatic leukaemia [25] and this is usually done by immunohistochemistry.

Pathologically, most specimens show sheets or cords of cells, undifferentiated or epithelial-like, with absent keratinisation; the common feature being a dense lymphoid infiltrate [8]. The use of immunohistochemistry is helpful in distinguishing LELC lesions, which differ in terms of prognosis and treatment. In LELC, tumour cells always express cytokeratin and EMA (Epithelial Membrane Antigen). Lymphomas on the other hand are negative for cytokeratin. The lymphoid cells of the stroma-reaction are in the majority of phenotype T: CD3 +, CD8 + mixed with some B lymphocytes [15].

As the tumour is rare, oncological treatment has not been clearly defined, yet. However, according to the literature review, most cases undergo mastectomy or lumpectomy with or without sentinel node biopsy [3]. Postoperative chemotherapy has been mentioned using cyclophosphamide and doxorubicin in many case reports although the exact role of systemic chemotherapy is unclear considering the low potential for metastasis. Radiotherapy has also been given as an adjuvant treatment [8,25].

As per multiple reported cases, LELC has been found to have a good prognosis with only 2-3 cases of nodal metastasis reported and no distant metastasis. Tumour-free five-year survival is almost 100% [26,14].

**Figure 7:**
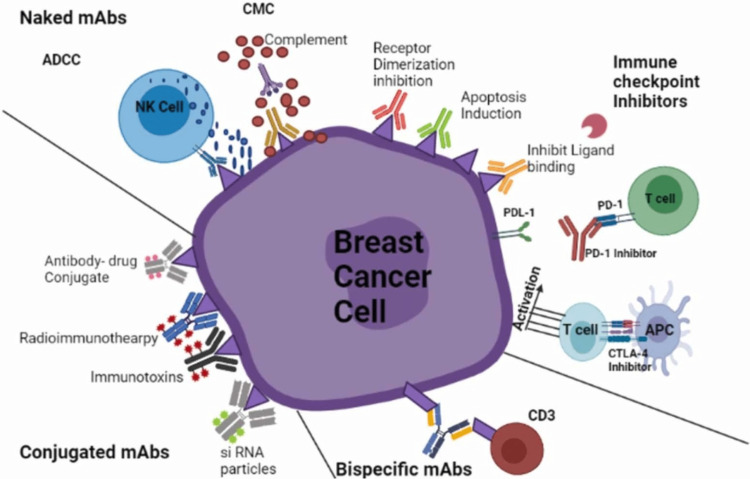
Different aspects of the use of monoclonal antibodies in breast cancers. mAbs: monoclonal antibodies

In the most recent advances regarding breast cancer treatment, monoclonal antibodies come to the fore. They have been variously used in breast cancers to target cancer cell lysis or to achieve delivery of chemotherapy drugs into specific cells, inhibiting cell growth or sometimes immunomodulation (Figure 7) [27].

In breast cancer, they have an important use in triple-negative cancers [28] and to improve the prognosis in Her receptor-positive cancers [29]. Case reports mention their use in lymphoepithelioma of salivary glands and lungs [30]. However, no case reports have mentioned so far its use specifically in lymphoepithelioma of the breast. Perhaps the usual good prognosis seen in the limited number of cases does not merit their use.

## Conclusions

Lymphoepithelial carcinoma can be an enigmatic tumour to diagnose and can be found in multiple sites in the body, breast being one of them although rare. However, once diagnosed appropriately, the treatment plan is straightforward like any other breast cancer, and it has a favourable prognosis. The entity is still rare and under evaluation and as yet no references for the treatment of breast lymphoepithelioma by monoclonal antibodies were found in this review.

## References

[1] Kumar S, Kumar D (1994). Lymphoepithelioma-like carcinoma of the breast. Mod Pathol.

[2] Nio Y, Tsuboi K, Tamaoki M, Tamaoki M, Maruyama R (2012). Lymphoepithelioma-like carcinoma of the breast: a case report with a special analysis of an association with human papilloma virus. Anticancer Res.

[3] Nankin NL, Gondusky CJ, Abasolo PA, Kalantari BN (2015). Lymphoepithelioma-like carcinoma of the breast. Radiol Case Rep.

[4] Cristina S, Boldorini R, Brustia F, Monga G (2000). Lymphoepithelioma-like carcinoma of the breast. An unusual pattern of infiltrating lobular carcinoma. Virchows Arch.

[5] Dadmanesh F, Peterse JL, Sapino A, Fonelli A, Eusebi V (2001). Lymphoepithelioma-like carcinoma of the breast: lack of evidence of Epstein-Barr virus infection. Histopathology.

[6] Naidoo P, Chetty R (2001). Lymphoepithelioma-like carcinoma of the breast with associated sclerosing lymphocytic lobulitis. Arch Pathol Lab Med.

[7] Peştereli HE, Erdogan O, Kaya R, Karaveli FS (2002). Lymphoepithelioma-like carcinoma of the breast. APMIS.

[8] Sanati S, Ayala AG, Middleton LP (2004). Lymphoepithelioma-like carcinoma of the breast: report of a case mimicking lymphoma. Ann Diagn Pathol.

[9] Ilvan S, Celik V, Ulker Akyildiz E, Senel Bese N, Ramazanoglu R, Calay Z (2004). Lymphoepithelioma-like carcinoma of the breast: is it a distinct entity?: Clinicopathological evaluation of two cases and review of the literature. Breast.

[10] Kurose A, Ichinohasama R, Kanno H, Kobayashi T, Ishida M, Nishinari N, Sawai T (2005). Lymphoepithelioma-like carcinoma of the breast. Report of a case with the first electron microscopic study and review of the literature. Virchows Arch.

[11] Saleh R, DaCamara P, Radhi J, Boutross-Tadross O (2005). Lymphoepithelioma-like carcinoma of the breast mimicking nodular sclerosing Hodgkin's lymphoma. Breast J.

[12] Kulka J, Kovalszky I, Svastics E, Berta M, Füle T (2008). Lymphoepithelioma-like carcinoma of the breast: not Epstein-Barr virus-, but human papilloma virus-positive. Hum Pathol.

[13] O'Sullivan-Mejia E, Idowu MO, Davis Masssey H, Cardenosa G, Grimes MM (2009). Lymphoepithelioma-like carcinoma of the breast: diagnosis by core needle biopsy. Breast J.

[14] Jeong AK, Park SB, Kim YM (2010). Lymphoepithelioma-like carcinoma of the breast. J Ultrasound Med.

[15] Dinniwell R, Hanna WM, Mashhour M, Saad RS, Czarnota GJ (2012). Lymphoepithelioma-like carcinoma of the breast: a diagnostic and therapeutic challenge. Curr Oncol.

[16] Suzuki I, Chakkabat P, Goicochea L, Campassi C, Chumsri S (2014). Lymphoepithelioma-like carcinoma of the breast presenting as breast abscess. World J Clin Oncol.

[17] Trihia H, Siatra H, Gklisty H, Diamantopoulos P, Arapantoni-Dadiotis P, Kalogerakos K (2012). Lymphoepithelioma-like carcinoma of the breast: cytological and histological features and review of the literature. Acta Cytol.

[18] Abdou AG, Asaad NY (2015). Lymphoepithelioma-like carcinoma of the breast: cytological, histological, and immunohistochemical characteristics. Diagn Cytopathol.

[19] Top OE, Vardar E, Yagci A, Deniz S, Ozturk R, Zengel B (2014). Lymphoepithelioma-like carcinoma of the breast: a case report. J Breast Health.

[20] Sato A, Kawasaki T, Abo-Yashima A (2017). Cytological features of lymphoepithelioma-like carcinoma of the breast. Cytopathology.

[21] Herrera-Goepfert R, Caro-Sánchez C, Maafs-Molina E (2016). Lymphoepithelioma-like carcinoma of the breast: a singular morphological pattern with an expected outcome. Austin J Clin Case Rep.

[22] Shet T, Pai T, Shetty O, Desai S (2016). Lymphoepithelioma-like carcinoma of breast-evaluation for Epstein-Barr virus-encoded RNA, human papillomavirus, and markers of basal cell differentiation. Ann Diagn Pathol.

[23] Aridi T, Fawwaz M, Kassab A (2018). Lymphoepithelioma-like carcinoma of the breast: a case report unveiling several clinical and histopathological challenges. Case Rep Surg.

[24] Lespagnard L, Cochaux P, Larsimont D, Degeyter M, Velu T, Heimann R (1995). Absence of Epstein-Barr virus in medullary carcinoma of the breast as demonstrated by immunophenotyping, in situ hybridization and polymerase chain reaction. Am J Clin Pathol.

[25] Salehiazar S, Huang H, Aghighi M, Venegas R (2022). Lymphoepithelioma-like carcinoma of the breast: a case report of a rare type of invasive carcinoma. Cureus.

[26] Ho JC, Wong MP, Lam WK (2006). Lymphoepithelioma-like carcinoma of the lung. Respirology.

[27] Behl A, Wani ZA, Das NN, Parmar VS, Len C, Malhotra S, Chhillar AK (2023). Monoclonal antibodies in breast cancer: a critical appraisal. Crit Rev Oncol Hematol.

[28] Wesolowski J, Tankiewicz-Kwedlo A, Pawlak D (2022). Modern immunotherapy in the treatment of triple-negative breast cancer. Cancers (Basel).

[29] Swain SM, Shastry M, Hamilton E (2023). Targeting HER2-positive breast cancer: advances and future directions. Nat Rev Drug Discov.

[30] Xiao Y, He J, Luo S (2022). Comparison of immunotherapy, chemotherapy, and chemoimmunotherapy in advanced pulmonary lymphoepithelioma-like carcinoma: a retrospective study. Front Oncol.

